# Supplementary material for: Dynamics of nevus development implicate cell cooperation in the growth arrest of transformed melanocytes
Source: eLife. 2020 Oct 13;9:e61026. doi: 10.7554/eLife.61026 (PMC7553774; doi:10.7554/eLife.61026)
Supplement: Transparent reporting form [file elife-61026-transrepform.docx]

***eLife’s* transparent reporting form**

We encourage authors to provide detailed information *within their submission* to facilitate the interpretation and replication of experiments. Authors can upload supporting documentation to indicate the use of appropriate reporting guidelines for health-related research (see [EQUATOR Network](http://www.equator-network.org/%20)), life science research (see the [BioSharing Information Resource](https://biosharing.org/)), or the [ARRIVE guidelines](http://www.plosbiology.org/article/info:doi/10.1371/journal.pbio.1000412) for reporting work involving animal research. Where applicable, authors should refer to any relevant reporting standards documents in this form.

If you have any questions, please consult our Journal Policies and/or contact us: [editorial@elifesciences.org](mailto:editorial@elifesciences.org).

**Sample-size estimation**

- You should state whether an appropriate sample size was computed when the study was being designed
- You should state the statistical method of sample size computation and any required assumptions
- If no explicit power analysis was used, you should describe how you decided what sample (replicate) size (number) to use

Please outline where this information can be found within the submission (e.g., sections or figure legends), or explain why this information doesn’t apply to your submission:

Sample size here refers both to samples for scRNAseq (individual animals, cells), and samples for measurements of nevus/nest size distributions (individual animals, nevi).  In the case of scRNA seq initial sample sizes were influenced by the limits of the sequencing methodology (number of cells that could be handled).  In all cases the adequacy of sample sizes was verified post hoc by comparing relative (normalized) standard errors of the means (or log-transformed means) of observed data with the differences between means for different cell types or conditions.

**Replicates**

- You should report how often each experiment was performed
- You should include a definition of biological versus technical replication
- The data obtained should be provided and sufficient information should be provided to indicate the number of independent biological and/or technical replicates
- If you encountered any outliers, you should describe how these were handled
- Criteria for exclusion/inclusion of data should be clearly stated
- High-throughput sequence data should be uploaded before submission, with a private link for reviewers provided (these are available from both GEO and ArrayExpress)

Please outline where this information can be found within the submission (e.g., sections or figure legends), or explain why this information doesn’t apply to your submission:

- The single cell data utilizes two mice for each genotype (Braf^WT^, Braf^V600E^) at P30 and three mice for each genotype (Braf^WT^, Braf^V600E^) at P50 for a total of 10 mice. This information can be found in the legend of Figure 2 and in the text under experimental design. The single cell data can be accessed through GEO accession number GSE154679 (Reviewer token yrinkgooxfgxtuz).
- Three mice were used to determine the nevus size distributions (found in the legend in Figure 3E) from 768 nevi.
- Simulations in figure 3 were a result from a minimum of 20,000 runs (outlined in figure 3 legend).
- CompuCell3D simulations were a result of 100 independent runs as stated in figure 5C-D legend.
- Five human nevi were utilized in our study (outlined under section “Quantification of nevus and nest size and cell content”).
- Quantification of nest radii at P21 (10 mice, 221 nests) and P50 (18 mice, 428 nests) can be found in the legend of Figure 4 supplement 1.

**Statistical reporting**

- Statistical analysis methods should be described and justified
- Raw data should be presented in figures whenever informative to do so (typically when N per group is less than 10)
- For each experiment, you should identify the statistical tests used, exact values of N, definitions of center, methods of multiple test correction, and dispersion and precision measures (e.g., mean, median, SD, SEM, confidence intervals; and, for the major substantive results, a measure of effect size (e.g., Pearson's r, Cohen's d)
- Report exact p-values wherever possible alongside the summary statistics and 95% confidence intervals. These should be reported for all key questions and not only when the p-value is less than 0.05.

Please outline where this information can be found within the submission (e.g., sections or figure legends), or explain why this information doesn’t apply to your submission:

- Statistical analyses are described in the Experimental design section under the subsection “Statistical Analysis.”
- Figure legends contain the median for appropriate data (i.e. Figure 4E, I, 5B, C, Figure S4,

(For large datasets, or papers with a very large number of statistical tests, you may upload a single table file with tests, Ns, etc., with reference to sections in the manuscript.)

**Group allocation**

- Indicate how samples were allocated into experimental groups (in the case of clinical studies, please specify allocation to treatment method); if randomization was used, please also state if restricted randomization was applied
- Indicate if masking was used during group allocation, data collection and/or data analysis

Please outline where this information can be found within the submission (e.g., sections or figure legends), or explain why this information doesn’t apply to your submission:

- For nevus/nest size distributions, samples were grouped based on age. The details can be found in the figure legends.
- For single cell RNA sequencing, samples were grouped based on either age and genotype. Details can be found in the legend for Figure 2 and in the section titled “Cell Isolation for Single Cell RNA Sequencing”

**Additional data files (“source data”)**

- We encourage you to upload relevant additional data files, such as numerical data that are represented as a graph in a figure, or as a summary table
- Where provided, these should be in the most useful format, and they can be uploaded as “Source data” files linked to a main figure or table
- Include model definition files including the full list of parameters used
- Include code used for data analysis (e.g., R, MatLab)
- Avoid stating that data files are “available upon request”

Please indicate the figures or tables for which source data files have been provided:

- Parameters for CompuCell3D modeling can be found in Data S2.
- We include a “Mathematical Supplement” that describe equations and biological processes described in the manuscript.
